# *Cryptosporidium* species and subtypes identified in human domestic cases through the national microbiological surveillance programme in Sweden from 2018 to 2022

**DOI:** 10.1186/s12879-024-09049-x

**Published:** 2024-01-30

**Authors:** Ioana Bujila, Karin Troell, Jessica Ögren, Anette Hansen, Gustav Killander, Lady Agudelo, Marianne Lebbad, Jessica Beser

**Affiliations:** 1 Department of Microbiology, Unit of Parasitology, Public Health Agency of Sweden, Solna, Sweden; 2 Department of Microbiology, National Veterinary Agency, Uppsala, Sweden; 3 Norwegian Veterinary Institute, Ås, Norway; 4 Division of Clinical Microbiology, Laboratory Medicine, Jönköping County, Jönköping Sweden; 5 Department of Clinical and Experimental Medicine, Linköping University, Linköping, Sweden; 6 Department of Communicable Disease Control and Health Protection, Unit of Zoonoses and Antibiotic Resistance, Public Health Agency of Sweden, Solna, Sweden

**Keywords:** *Cryptosporidium*, Surveillance, Molecular typing, Protozoa, Epidemiology, Zoonosis

## Abstract

**Background:**

The intestinal protozoan parasite *Cryptosporidium* is an important cause of diarrheal disease worldwide. A national microbiological surveillance programme was implemented in Sweden in 2018 in order to increase knowledge of the molecular epidemiology of human cryptosporidiosis to better understand transmission patterns and potential zoonotic sources. This article summarises the results of the first five years of the surveillance programme.

**Methods:**

*Cryptosporidium*-positive faecal and DNA samples from domestically acquired infections were collected from clinical microbiological laboratories in Sweden. Species and subtype determination was performed using 60 kDa glycoprotein and/or small subunit ribosomal RNA gene analysis.

**Results:**

Between 2018 and 2022, 1654 samples were analysed and 11 different species were identified: *C. parvum* (*n* = 1412), *C. mortiferum* (*n* = 59), *C. hominis* (*n* = 56), *C. erinacei* (*n* = 11), *C. cuniculus* (*n* = 5), *C. meleagridis* (*n* = 3), *C. equi* (*n* = 2), *C. ubiquitum* (*n* = 2), and one each of *C. canis, C. ditrichi* and *C. felis*. Subtyping revealed seven subtype families of *C. parvum* (new subtype families IIy and IIz) and 69 different subtypes (11 new subtypes). The most common *C. parvum* subtypes were IIdA22G1c, IIdA24G1, IIdA15G2R1 and IIaA16G1R1b. For *C. hominis*, four different subtype families and nine different subtypes (two new subtypes) were identified. For additional species, two new subtype families (IIIk and VId) and nine new subtypes were identified. All successfully subtyped *C. mortiferum* cases were subtype XIVaA20G2T1, confirming previous findings in Sweden. Several outbreaks were identified of which the majority were foodborne and a few were due to direct contact with infected animals.

**Conclusion:**

Infection with *C. parvum* is the leading cause of human cryptosporidiosis acquired in Sweden, where more than 90% of domestic cases are caused by this zoonotic species and only a small proportion of cases are due to infection with other species. The rodent-associated *C. mortiferum* is considered an emerging zoonotic species in Sweden and the number of domestically acquired human cases has surpassed that of infection with *C. hominis.* A high diversity of species and subtypes, as well as diversity within the same subtype, was detected. Also, cryptosporidiosis appears to affect adults to a great extent in Sweden.

**Supplementary Information:**

The online version contains supplementary material available at 10.1186/s12879-024-09049-x.

## Introduction

*Cryptosporidium* spp. are protozoan parasites that can cause disease in both humans and animals. To date, 21 species and two genotypes have been reported in humans [1–3]. The majority of human cases are caused by either *Cryptosporidium parvum* or *Cryptosporidium hominis*, which are responsible for more than 95% of infections in humans worldwide [2, 4]. These two species differ in host range and global distribution whereby infection with *C. parvum* has a broader host range including ruminants, primates, equine animals and rodents, whereas *C. hominis* is mainly restricted to humans and other primates and equine animals [4]. Other species that cause infection in humans include *Cryptosporidium mortiferum*, previously known as *Cryptosporidium* chipmunk genotype I, *Cryptosporidium meleagridis, Cryptosporidium cuniculus, Cryptosporidium ubiquitum, Cryptosporidium canis, Cryptosporidium felis, Cryptosporidium viatorum* [3, 4] and the relatively recently described *Cryptosporidium ditrichi* [5].

Cryptosporidiosis in humans usually presents with self-limiting diarrhoea, but the disease can be severe, especially in immunocompromised individuals [6]. However, persisting long-term gastrointestinal symptoms such as diarrhoea, abdominal pain, nausea, fatigue and headache have been reported as well as a possible connection to the onset of inflammatory bowel disease and microscopic colitis [7–12]. Thus far, no effective treatment or vaccines against cryptosporidiosis have been developed. Control depends on understanding the dynamics of infection, in which molecular methods play a crucial role [2].

In Sweden, cryptosporidiosis has been a notifiable disease in humans since 2004. The incidence of cryptosporidiosis has increased from 0.8 cases/100,000 inhabitants in 2005 to 6.8 cases/100,000 inhabitants in 2022. The increase is attributable to, for example the development of better diagnostic tools such as multiplex PCR assays [13], as well as increased awareness and knowledge of cryptosporidiosis due to two large waterborne outbreaks that occurred in Sweden in 2010 and 2011 which affected approximately 45,000 residents [14, 15]. Several foodborne outbreaks and outbreaks associated with animal contact, including one caused by *C. mortiferum* in 2019, have also been reported [16–21].

In order to better understand the molecular epidemiology of human cryptosporidiosis in Sweden, a study was conducted from 2013 to 2014 involving clinical laboratories from various counties [22], followed by the implementation of a national microbiological surveillance programme for *Cryptosporidium* in 2018 by the Public Health Agency of Sweden (PHAS).

This article describes the results obtained through the national microbiological surveillance programme for *Cryptosporidium* in Sweden between 2018 and 2022.

## Materials and methods

### National microbiological surveillance programme

*Cryptosporidium* positive samples of domestically acquired infection were asked to be sent to the PHAS for typing. The primary diagnostics of cryptosporidiosis in Sweden is done by local clinical microbiological laboratories by multiplex real-time PCR and/or to a lesser extent by light microscopy, using modified Ziehl-Neelsen staining. In 2018 and 2019, samples were typed throughout the year, while in 2020 and onwards, the surveillance period and typing were changed to include samples from 1 August to 30 November.

Each *Cryptosporidium* positive sample included information about the age, sex and geographical location of the patient.

### Molecular investigations

DNA from faecal samples was extracted using a magLEAD 12gC instrument supplied with magDEA DX MV reagents (Precision System Science Co Ltd., Chiba, Japan). All extractions were performed according to the manufacturer’s instructions. Prior to extraction, oocyst disruption was achieved by bead beating using a Bullet Blender (Techtum, Sweden). Determination of species and subtypes was done by amplification of the 60 kDa glycoprotein (*gp60*) gene using primers as published by Alves et al. [23] followed by bi-directional sequencing of the PCR amplicons. Unsuccessful *gp60* amplification and the detection of novel subtype families were followed by amplification of the small subunit rRNA (ssu rRNA) gene by PCR, and bi-directional sequencing of the PCR amplicon [24, 25]. *C. mortiferum* and *C. meleagridis* samples were subtyped using primers, as described in Guo et al. and Stensvold et al., respectively [26, 27]. Editing and analysis of sequences was done using CLC Main Workbench (Qiagen, Aarhus, Denmark, version 8). The obtained sequences were compared to isolates in the GenBank database using the Basic Local Alignment Search Tool (BLAST; NCBI www.ncbi.nlm.nih.gov/blast/BLAST.cgi).

In this article, a subtype is defined as a *gp60* sequence that has a given unique subtype name. Smaller variations in the conserved non-repetitive region, that have not already been given a unique name, are considered variants of an existing subtype. These sequences are referred to by the subtype name and corresponding GenBank accession number (acc. no.) with 100% identity.

### Phylogenetic analysis

Phylogenetic analysis was performed on the newly generated *gp60* DNA sequences, as well as sequences from known *Cryptosporidium* species and subtypes. A phylogenetic tree was generated using the neighbor-joining method based on Kimura’s 2-parameter model [28]. To estimate robustness, bootstrap proportions were computed after 1000 replications. Evolutionary analyses were conducted in MEGA XI (https://www.megasoftware.net/ accessed on 2 September 2023).

### GenBank

Representative nucleotide sequences that were generated were submitted to the GenBank database under the following accession numbers: OL598537-OL598578, OM146534-OM146540, OM160950-OM160951, OR491772-OR491782 and OR790948-OR790949.

## Results

### Case demographics

**Fig. 1:**
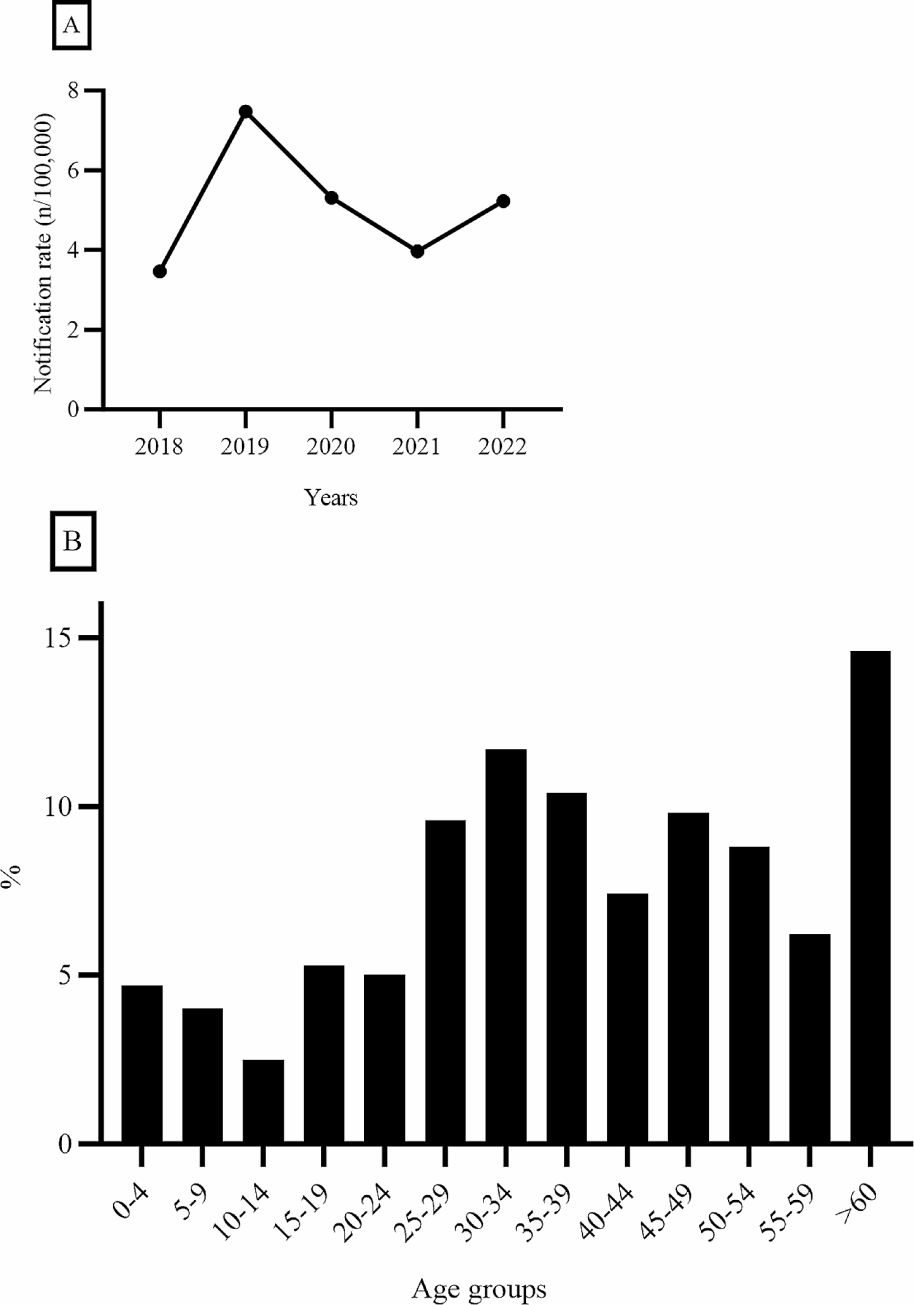
Notification rate of domestic cryptosporidiosis cases from 2018 to 2022 (**A**) and distribution of the submitted samples based on age groups (**B**)

Between 2018 and 2022, 3684 cryptosporidiosis cases were reported to the national mandatory notifications system (SmiNet), of which 2639 (72%) were domestic, 950 (26%) had travel history and 95 had no information. The notification rate of domestic cryptosporidiosis from 2018 to 2022 is shown in Fig. 1A. During this period, 1850 samples were sent to the PHAS for typing and 1654 samples were further analysed. Not all samples sent for typing during the national outbreak in 2019 were analysed since a sample selection had to be made. Also, not all samples were successfully amplified. The majority of cases were annually reported between July and December, peaking in 2019 and 2022, due to national outbreaks. The age distribution of the patients is shown in Fig. 1B.

Also, the notification rate of domestic cryptosporidiosis cases in different age groups compared to the average notification rate in the EU/EEA is shown in Supp. Figure 1.

From the submitted samples, the age range was 3 months–98 years with a mean age of 38 years. 57% of the samples were from women and 43% were from men.

### *Cryptosporidium* species

Species determination was successful for 94% (*n* = 1553) of the samples and 11 different species were identified. *C. parvum* was detected in 91% of the samples and was thus the most common species (*n* = 1412). The second most common species was *C. mortiferum* (4%; *n* = 59) followed by *C. hominis* (3.6%; *n* = 56). However, no *C. hominis* positive samples were detected in 2020. Other less common species detected were *C. erinacei* (*n* = 11), *C. cuniculus* (*n* = 5), *C. meleagridis* (*n* = 3), *C. equi* (*n* = 2), previously known as *Cryptosporidium* horse genotype, *C. ubiquitum* (*n* = 2), as well as one case each of *C. canis, C. ditrichi* and *C. felis* (Table 1).

**Table 1:** *Cryptosporidium* spp. identified in Sweden from 2018 to 2022

Species	N (%)
*C. parvum*	1412 (91)
*C. mortiferum* ^1,2^	59 (4)
*C. hominis*	56 (3.7)
*C. erinacei*	11 (< 1)
*C. cuniculus*	5 (< 1)
*C. meleagridis*	3 (< 1)
*C. equi* ^3^	2 (< 1)
*C. ubiquitum*	2 (< 1)
*C. canis*	1 (< 1)
*C. ditrichi* ^4^	1 (< 1)
*C. felis*	1 (< 1)

^1^ Previously known as *Cryptosporidium* chipmunk genotype I

^2^ 20 cases were published by Bujila et al., 2021 [18]

^3^ Previously known as *Cryptosporidium* horse genotype

^4^ Published by Beser et al., 2020 [5]

All 21 counties in Sweden reported cases of *C. parvum*, while only ten reported cases of *C. hominis* and 13 reported cases of *C. mortiferum*.

**Fig. 2:**
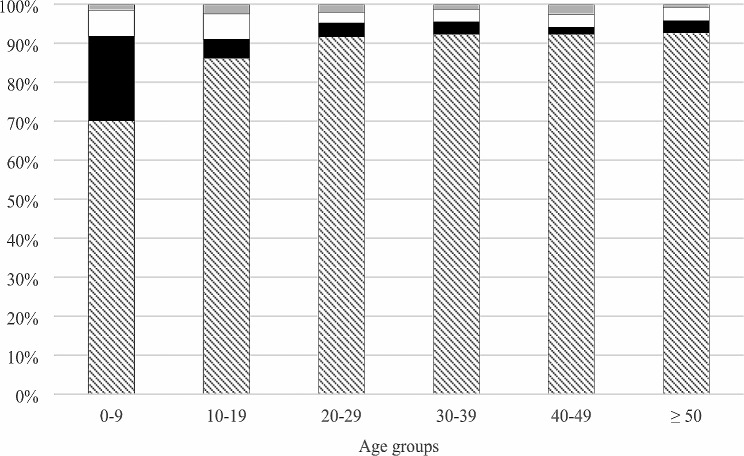
Distribution of *Cryptosporidium* species in different age groups. *C. parvum*: striped, *C. hominis*: black, *C. mortiferum*: white and other species: light grey

The distribution of different *Cryptosporidium* species in different age groups is shown in Fig. 2.

Infection with *C. parvum* was more common in women (58%) than in men (41%) while infection with *C. hominis* and *C. mortiferum* was more common in men (56% in men; 44% in women and 53% in men; 43% in women, respectively).

### Molecular characterisation of *Cryptosporidium parvum*

Seven different *C. parvum* subtype families (IIa, IIc, IId, IIe, IIl, IIy and IIz) were detected, of which IIy and IIz are new subtype families. The most common subtype families were IIa (*n* = 692) and IId (*n* = 663). Within the IIa subtype family, 39 different subtypes were detected, of which IIaA15G2R1 (15% of *C. parvum* cases), IIaA16G1R1b (12% of the *C. parvum* cases) and IIaA17G1R1c (5% of *C. parvum* cases) were the most common subtypes (Table 2).

**Table 2:** *C. parvum* subtype families and subtypes identified in Sweden from 2018 to 2022

Subtype family (n)	Subtype (n)^1,2^	GenBank acc. no.^3^	Comment^4,5^
**IIa (692)**	IIaA12G1R1r1 (1)	**OR491776**	New subtype 2018
	IIaA13G1R1 (14)	GU111578	Only detected in Halland County
	IIaA13G2R1 (3)	KU852706	-
	IIaA13R1 (8)	KU852702	-
	IIaA14R1 (25)	JX183797	**Associated with a local outbreak 2022 (** *n* ** = 4)**
	IIaA14G1R1 (14)	KF128737	-
	IIaA14G1R1r1 (14)	KU852703	**Associated with a local outbreak 2022 (** *n* ** = 2)**
	IIaA15G1R1 (2)	**OL598550**	2018
	IIaA15G1R1 (1)	**OL598568**	2019
	IIaA15G1R1 (2)	JF727794	2021
	IIaA15G1R1_variant (5)	KU852704	2022
	IIaA15G1R1r1_variant (1)	**OR491775**	New subtype 2022
	**IIaA15G2R1 (216)**	AF164490	**Associated with a local outbreak in 2022 (** *n* ** = 2) and a national outbreak in 2022 (** *n* ** = 107)**
	**IIaA16G1R1b (163)**	EU647727	**Associated with a local outbreak in 2019 (** *n* ** = 4) and 2021 (** *n* ** = 1)**
	IIaA16G1R1b_variant (7)	KT895368	-
	IIaA16G2R1 (4)	DQ192505	-
	IIaA16G3R1 (1)	DQ192506	2018
	IIaA17G1R1 (4)	GQ983359	-
	IIaA17G1R1c (71)	JX183801	**Associated with a local outbreak in 2020 (** *n* ** = 7)**
	IIaA17G1R1c_variant (8)	AF403168	-
	IIaA17G2R1 (4)	AF164493	-
	IIaA17G2R1_variant (1)	**OR491772**	New subtype 2020
	IIaA17R1 (4)	JX183800	-
	IIaA18G1R1b (11)	KF289039	-
	IIaA18G1R1b_variant (18)	KT895369	**Associated with a local outbreak in 2021 (** *n* ** = 3)**
	IIaA18G1R1d (5)	JX183803	Only detected in Uppsala County
	IIaA18G2R1 (1)	DQ630515	2018
	IIaA18G3R1 (1)	DQ192508	2019
	IIaA18G5R1 (1)	MF142037	2019
	IIaA18R1 (1)	KU852705	2022
	IIaA19G1R1 (2)	KC679056	2022
	IIaA19G2R1 (1)	DQ630514	2021
	IIaA19R1 (3)	**OL598555**	New subtype
	IIaA20G1R1 (20)	JX183804	-
	IIaA20G1R1 (1)	**OL598549**	2018
	IIaA20G1R1 (1)	**OL598553**	2019
	IIaA20G1R1_variant (1)	**OL598567**	New subtype 2019
	IIaA20R1 (1)	JQ028867	2021
	IIaA21G1R1 (27)	FJ917373	-
	IIaA21R1 (1)	**OL598556**	2019
	IIaA22G1R1 (14)	JX183806	-
	IIaA22G1R1 (1)	**OL598539**	2018
	IIaA23G1R1 (4)	KC995126	-
	IIaA24G1R1 (4)	**OR491777**	Only detected in Halland County May in 2019
**IIc (4)**	IIcA5G3j (1)	HQ005749	2022
	IIcA5G3o (3)	KU670812	Only detected in Halland County in 2021
**IId (663)**	IIdA7 (1)	**OR491780**	New subtype 2022
	IIdA16G1 (13)	JX183808	-
	IIdA16G1b (9)^6^	FJ17372	-
	IIdA17G1 (6)	KU852708	-
	IIdA18G1 (4)	KU852709	-
	IIdA18G1 (2)	EF576975	-
	IIdA18G1 (1)	MH796389	-
	IIdA19G1 (18)	DQ280496	**Associated with a local outbreak in 2022 (** *n* ** = 1)**
	IIdA19G1 (4)	**OR491778**	-
	IIdA20G1 (3)	**OL598548**	-
	IIdA20G1b (2)	AY738185	-
	IIdA20G1d (1)	AY738186	2018
	IIdA20G1e (72)	JQ028866	**Associated with a local outbreak in 2019 (** *n* ** = 2)**
	IIdA21G1 (12)	DQ280497	-
	IIdA21G1 (48)	**OL598545**	**Associated with local outbreaks in 2019 (** *n* ** = 2), 2020 (** *n* ** = 4) and 2021 (** *n* ** = 1)**
	IIdA22G1 (15)	AY166806	**Associated with a local outbreak in 2019 (** *n* ** = 4)**
	IIdA22G1 (5)	KR349103	-
	IIdA22G1 (3)	**OR491774**	-
	**IIdA22G1c (211)**	FJ917374	**Associated with a national outbreak 2019 (** *n* ** = 122) and a local outbreak 2022 (** *n* ** = 2)**
	IIdA23G1 (17)	EU868625	**Associated with a local outbreak in 2021 (** *n* ** = 12)**
	IIdA23G1 (12)	FJ917376	-
	IIdA23G1 (2)	KR349095	-
	IIdA23G1 (1)	KP997136	2021
	**IIdA24G1 (160)**	JQ028865	**Associated with a national outbreak in 2019 (** *n* ** = 65)**
	IIdA24G1 (2)	EU549714	2018
	IIdA24G1c (1)	JX183810	2018
	IIdA25G1 (23)	JX043492	-
	IIdA25G1 (1)	**OL598537**	2018
	IIdA26G1b (1)	JX183811	2019
	IIdA27G1 (7)	**OL598546**	Only detected in Östergötland County
	IIdA27G1_variant (2)	**OL598571**	New subtype 2020
	IIdA28G1 (4)	**OL598551**	New subtype
**IIe (8)**	IIeA11G1 (5)	**OL598540**	Only detected in Stockholm County
	IIeA11G1 (1)	**OL598561**	2019
	IIeA11G1 (1)	**OL598572**	2022
	IIeA14G1 (1)	**OR491779**	New subtype 2019
**IIl (3)**	IIlA16R2 (2)	AM937007	-
	IIlA17R2 (1)	**OL598577**	2021
**IIy** ^7^ **(2)**	IIyA23G1R1 (2)	**OL598564**	New subtype family
**IIz** ^7^ **(2)**	IIzA14R2 (2)	**OL598569**	New subtype family

^1^ The four most common *C. parvum* subtypes are indicated in bold

^2^*gp60* PCR negative (*n* = 38)

^3^ Sequences submitted to GenBank are indicated in bold. Non bold accession numbers refer to reference sequences from GenBank

^4^ Association with outbreak(s) are indicated in bold

^5^ When a county is stated it refers to subtypes (n = > 1) that have only been detected in one county

^6^ One sample of this subtype was part of an investigation of zoonotic transmission published by Bujila et al., 2021 [18]

^7^ rRNA sequences have been deposited in GenBank with acc. no. OM146538 and OM146539, respectively

Within subtype family IId, 22 different subtypes were detected. The most common IId subtypes were IIdA22G1c (15% of *C. parvum* cases), IIdA24G1 (11% of *C. parvum* cases) and IIdA20G1e (5% of *C. parvum* cases) (Table 2).

**Fig. 3:**
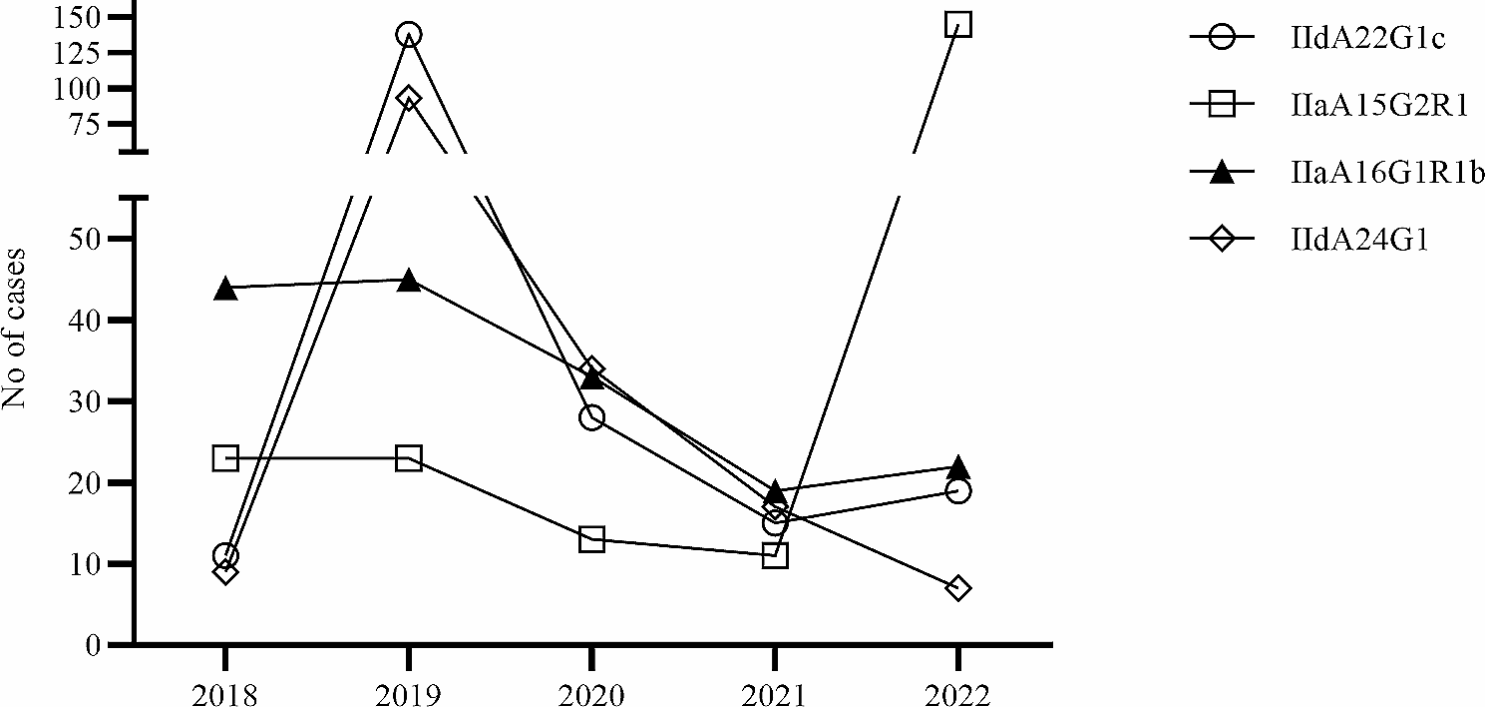
Distribution from 2018 to 2022 of the four most common *C. parvum* subtypes. Peaks in 2019 and 2022 are due to national outbreaks

The distribution of the four most common *C. parvum* subtypes is shown in Fig. 3.

The peaks of cases of IIdA22G1c and IIdA24G1 in 2019 and IIdA15G2R1 in 2022 are due to national outbreaks, whereas IIaA16G1R1b is annually one of the most common *C. parvum* subtypes reported but thus far has not caused any known major outbreaks between 2018 and 2022.

Of note, 11 new *C. parvum* subtypes were detected, IIa (*n* = 5), IId (*n* = 3), IIe (*n* = 1), IIy (*n* = 1) and IIz (*n* = 1). Subtype IIyA23G1R1 (OL598564) was detected in Uppsala County in 2019 and 2020 and subtype IIzA14R2 (OL598569) was detected in Västerbotten County in 2019 and 2021 and, as mentioned above, are new subtype families. The ssu rRNA sequences generated from three of the samples (OM146538) were identical to each other and to a sequence obtained from bamboo rats (MK956937). The sequences had one A to T substitution compared with *C. parvum* sequences commonly found in humans, cattle and other animals [29]. Further, a new subtype within the IId subtype family, IIdA7 (OR491780), which lacks TCG repeat(s) and only has 7 TCA repeats, was detected in 2022.

All subtypes and subtype variants are indicated by their respective GenBank acc. no in Table 2.

### Molecular characterisation of *Cryptosporidium hominis*

Four different subtype families (Ia, Ib, Id and Ie) and nine different subtypes were identified. The most common subtype was IbA10G2 (56% of *C. hominis* cases) followed by IbA12G3 (5% of *C. hominis* cases) (Table 3). In addition, two new subtypes were detected, IaA16R4 (OL598578) and IaA28R3 (OL598538).

**Table 3:** *C. hominis* subtype families and subtypes identified in Sweden from 2018 to 2022

Subtype family (n)	Subtype (total n)^1,2^	GenBank acc. no.^3^	Comment
**Ia (5)**	IaA11R3 (2)	MT009623	2019
	IaA16R4 (1)	**OL598578**	New subtype 2021
	IaA28R3 (1)	**OL598538**	New subtype 2018
	IaA30R3 (1)	**OR491782**	2018
**Ib (38)**	**IbA10G2 (32)**	AY262031	-
	IbA10G2 (1)	**OL598563**	2019
	**IbA12G3 (5)**	KY990894	2018
**Id (3)**	IdA17 (2)	KU852721	2022
	IdA21 (1)	FJ707316	2019
**Ie (8)**	**IeA11G3T3 (8)**	GU214354	-

^1^ The three most common *C. hominis* subtypes are indicated in bold

^2^*gp60* PCR negative (*n* = 2)

^3^ Sequences submitted to GenBank are indicated in bold. Non bold accession numbers refer to reference sequences from GenBank

### Molecular characterisation of additional species

Molecular characterisation of additional species is shown in Table 4. However, subtyping was either not successful or done for some of the cases. All cases of *C. mortiferum* in which subtyping was successful had subtype XIVaA20G2T1 and data concerning this species and subtype in Sweden are further presented by Bujila et al. [18]. Two new subtype families were detected, *C. meleagridis* IIIk with subtype IIIkA6R1 (OL598552) and *C. equi* VId with subtype VIdA10G1 (OL598562). The patient with IIIkA6R1 acquired the infection in 2018 and only contact with dog was reported. Genotype 2 was seen at the ssu rRNA locus (OM146536). The *C. equi* VId sample was further investigated at the actin, heat shock protein 70, *Cryptosporidium* oocyst wall protein, as well as ssu rRNA loci (OM160947, OM160948, OM160949 and OM146537, respectively). Phylogenetic analyses showed a high similarity with known *C. equi* sequences (data not shown). The patient acquired the infection in 2019 and no information on animal contact was obtained. The following additional new subtypes were detected: *C. erinacei* XIIIaA12R13 (OL598574), XIIIaA17R7 (OM160950), XIIIaA17R13 (OL598566), XIIIaA19R7 (OL598547), XIIIaA23R10 (OL598542), *C. cuniculus* VbA30R4 (OR491773) and *C. meleagridis* IIIaA20G4R (OL598544).

**Table 4:** Additional *Cryptosporidium* spp., subtype families and subtypes identified in Sweden from 2018 to 2022

Species (n)	Subtype families/subtypes (n)^1^	GenBank acc. no.^2^	Comment
***C. mortiferum*** **(59)** ^3^	XIVaA20G2T1 (32)	KU852739	Associated with a local outbreak in 2019 (*n* = 3)
***C. erinacei*** **(11)**	XIIIaA12R13 (1)	**OL598574**	New subtype 2020
	XIIIaA17R7 (1)	**OM160950**	New subtype 2020
	XIIIaA17R13 (1)	**OL598566**	New subtype 2019
	XIIIaA19R7 (2)	**OL598547**	New subtype 2018
	XIIIaA22R9 (1)	**OM160951**	2020
	XIIIaA22R11 (1)	**OL598565**	2019
	XIIIaA23R10 (1)	**OL598542**	New subtype 2018
***C. cuniculus*** **(5)**	VaA22 (3)	**OL598554**	-
	VbA29R4 (1)	GU097639	2021
	VbA30R4 (1)	**OR491773**	New subtype 2021
***C. meleagridis*** **(3)**	IIIaA20G4R1 (1)	**OL598544**	New subtype 2018
	IIIkA6R1 (1)^4^	**OL598552**	New subtype family 2018
***C. equi*** **(2)**	VIdA10G1 (1)^4^	OL598562	New subtype family 2019
	VIaA11G3 (1)	KU200960	-

^1^*gp60* PCR negative: *C. mortiferum* (*n* = 3), *C. erinacei* (*n* = 3) and *C. meleagridis* (*n* = 1)

^2^ Sequences submitted to GenBank are indicated in bold. Non bold accession numbers refer to reference sequences from GenBank

^3^ 20 cases published by Bujila et al., 2021 [18]

^4^ rRNA sequences have been deposited in GenBank with acc. no. OM146536 and OM146537, respectively

### Phylogenetic analysis

**Fig. 4:**
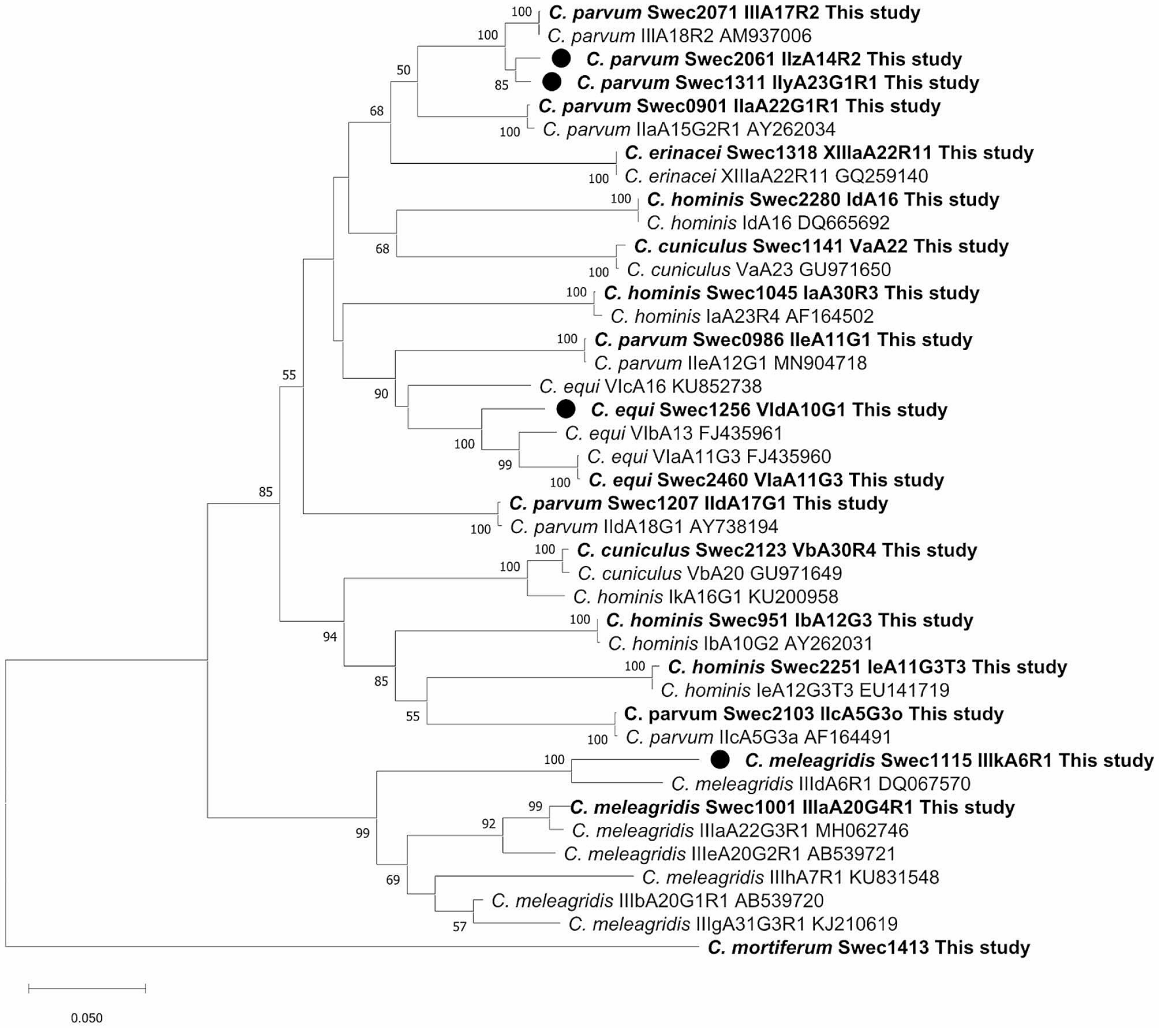
Phylogenetic relationships between partial *gp60 Cryptosporidium* DNA sequences obtained in the present study and sequences retrieved from the NCBI database. Each detected subtype family is represented by one study sample. The phylogenetic tree was constructed using the neighbor-joining method based on genetic distance calculated based on Kimura’s 2-parameter model as implemented in MEGA XI. The final dataset included 984 positions. Bootstrap values ≥ 50% from 1000 replicates are indicated at each node. New subtype families observed in this study are indicated by filled circles. All isolates from this study are indicated in bold

A phylogenetic tree that contained one representative *gp60* sequence from each subtype family detected within the national surveillance programme (*n* = 19) was constructed (Fig. 4). Sequences from established subtype families clustered with sequences from the national surveillance programme. The new *C. parvum* subtype families IIy and IIz clustered with each other and IIl. The new *C. meleagridis* subtype family IIIk clustered with IIId and the new *C. equi* subtype family VId with VIb and VIa.

### Outbreaks

Several outbreaks were identified between 2018 and 2022. The majority of outbreaks were foodborne and some were due to direct contact with infected animals. Of note, no outbreaks attributed to contaminated drinking or recreational water were detected. All outbreaks except one, which was caused by *C. mortiferum*, were caused by infection with *C. parvum* (Table 5).

**Table 5:** Outbreaks identified in Sweden from 2018 to 2022

Month/Year	Nr. of susp. cases	Nr. of typed cases	Comment	Species	Subtype
May 2019	12	4	Private event with 30 guests. Suspected contaminated salad	*C. parvum*	IIdA22G1
June 2019	10	4	Private midsummer event. Suspected contaminated salad	*C. parvum*	IIaA16G1R1b
October-December 2019	Unknown	122	National outbreak with unpasteurised juice as the source of contamination	*C. parvum* ^1^	IIdA22G1c
October-December 2019	Unknown	65	National outbreak with unknown source of contamination	*C. parvum* ^1^	IIdA24G1
October 2019	Unknown	3	Pre-school	*C. mortiferum* ^2^	XIVaA20G2T1
December 2019	Unknown	2	Christmas buffet with contaminated kale as the suspected source	*C. parvum*	IIdA21G1
December 2019	Unknown	2	Christmas buffet with contaminated kale as the suspected source	*C. parvum*	IIdA21G1
December 2019	Unknown	2	Christmas buffet with contaminated winter salad mix as the suspected source	*C. parvum*	IIdA20G1e
August 2020	7	7	Restaurant, suspected contaminated arugula	*C. parvum*	IIaA17G1R1c
December 2020	20	4	Elementary school, suspected contaminated kale	*C. parvum*	IIdA21G1_ OL598545
January 2021	23	18	Elderly home and two schools, contaminated kale	*C. parvum*	IIdA23G1 (*n* = 12), IIaA18G1R1b_variant (*n* = 3), IIaA16G1R1b (*n* = 1) and IIdA21G1 (*n* = 1)
February 2022	Unknown	4	Agricultural school	*C. parvum*	IIaA14R1
May 2022	3	2	Assisted living that also has various kinds of animals such as lambs	*C. parvum*	IIaA14G1R1r1
June 2022	4	2	School visit to farm with calves	*C. parvum*	IIaA15G2R1
September-October 2022	Unknown	107	National outbreak affecting 15 counties. Contaminated frisée salad as the suspected source	*C. parvum*	IIaA15G2R1
October-November 2022	4	1	Agricultural school	*C. parvum*	IIdA19G1
November 2022	75	2	Upper secondary school Contaminated salad buffet as the suspected source	*C. parvum*	IIdA22G1c

^1^ Manuscript in preparation

^2^ Published by Bujila et al., 2021 [18]

## Discussion

Cryptosporidiosis was the most common notifiable parasitic disease in Sweden in 2022 with a notification rate of 6.8/100,000 inhabitants. Regarding all notifiable diseases, it was the tenth most reported disease indicating that *Cryptosporidium* is relatively widespread in Sweden [30]. The notification rate of cryptosporidiosis in Sweden has increased since it became a notifiable disease in 2004 and is to be considered high in a European context: 6.8/100,000 inhabitants compared to the EU/EEA notification rate in 2022 of 3.2/100,000 inhabitants, as is also shown in Supp. Figure 1. However, notification of cryptosporidiosis in European countries and reporting to the European Surveillance System (TESSy) differs. In 2022, 24 out of the 30 EU/EEA countries reported cases, of which two countries reported zero cases [31]. The reporting of cryptosporidiosis was mandatory in 22 countries and no molecular typing data are collected and analysed at the EU/EEA level. With that in mind, the data presented in this report help to further understand the molecular epidemiology of cryptosporidiosis in Europe.

As previously mentioned, there are various reasons why the notification rate in Sweden has increased, such as better general knowledge, increased awareness, as well as the introduction of more sensitive diagnostic measures. The increasing use of multiplex assays detecting various agents, whereby clinicians do not need to specifically request diagnostics for *Cryptosporidium*, is also a likely cause of the increased notification rate. A study in Jönköping County in 2020 showed that cryptosporidiosis was underdiagnosed due to clinician’s choice of analysis. Cryptosporidiosis cases were detected in samples with suspected bacterial gastroenteritis but not in samples with suspected parasitic infection [13]. Similar results were found in a study of cryptosporidiosis in Denmark [32]. Thus the approach to detect cryptosporidiosis may need to change from suspicion of parasitic infection to more symptom-based diagnostics, as suggested by Ögren et al. and as a result will probably further increase the detection of cryptosporidiosis in Sweden and other countries [13].

Several reports have shown that cryptosporidiosis follows a bimodal seasonal pattern in Europe with an increase in spring and a peak in late summer/early autumn [33–36]. In Sweden, cryptosporidiosis increases in late summer and early autumn in line with observations from other European countries, but with no clear increase in cases during the spring.

Globally, cryptosporidiosis mainly affects children [33]. A characteristic of cryptosporidiosis acquired in Sweden compared to other EU/EEA countries is that the highest notification rate is not in children (0–4 years) [31]. Instead, adults (25–44 years) had the highest notification rate from 2018 onwards. Further, there is a higher proportion of *C. hominis* infections in children compared to other age groups.

Only a small proportion of domestically acquired cases are due to infection with *C. hominis*. During the COVID-19 pandemic in 2020 when travel abroad was restricted, no domestic cases of cryptosporidiosis caused by *C. hominis* were detected, suggesting that many infections with *C. hominis* are indeed contracted abroad and then occasionally causing secondary domestic transmission.

Infection with the rodent-associated *C. mortiferum* is considered an emerging zoonotic *Cryptosporidium* spp. in Sweden and the number of domestically acquired cases surpasses that of infection with *C. hominis* [18]. An important aspect regarding the detection of various *Cryptosporidium* spp. is the choice of PCR method. PCR is a highly sensitive method. However, this might not be the case for some PCRs designed to detect cryptosporidiosis. Several in-house and commercially available multiplex real-time PCR assays have been designed to detect infection by species belonging to the *C. parvum*/*C. hominis* complex. Hence, the sensitivity of species more distant from *C. parvum* and *C. hominis* is affected [37, 38]. It may give a bias in the number of detected species, as well as an underestimation in prevalence.

In accordance with the published data, the two most common *C. parvum* families in Sweden are IIa and IId. However, none of them are more predominant than the other. In industrialised countries, the *C. parvum* subtype IIaA15G2R1 is a dominant subtype in both humans and cattle [2]. This is also a relatively common subtype annually in Sweden. In 2022, it was responsible for a national foodborne outbreak. Table 2 shows other relatively common *C. parvum* subtypes. In summary, several *C. parvum* subtypes are commonly detected in Sweden. Further, many different subtypes and subtype variants of *C. parvum* were detected, suggesting a very high level of diversity. However, regarding the subtypes of *C. mortiferum*, there is a lack of diversity as all cases were subtype XIVaA20G2T1 [18]. This could possibly be attributed to the fact that to date only one natural host (red squirrel) has been detected in Sweden.

The most prevalent *C. hominis* subtype was IbA10G2, which is in accordance with published data [2]. Recent data have shown that IfA12G1R5 appears to have replaced IbA10G2 as the most common *C. hominis* subtype in the United States [39]. However, only one domestic case of this subtype has been detected in Sweden [22].

Outbreaks associated with drinking and recreational water (swimming pools) are still the main cause of outbreaks due to *Cryptosporidium* spp. in both Europe and the United States [33, 34]. Since the national microbiological surveillance programme for *Cryptosporidium* started in Sweden in 2018, no outbreaks due to contaminated water and/or infection with *C. hominis* have been detected. Instead, all detected outbreaks were associated with food, such as green leafy vegetables or direct contact with animals. This highlights how different kinds of food, particularly different types of lettuce are important vehicles of infection. In fact, several studies have shown contamination by *Giardia duodenalis* and *Cryptosporidium* spp. in green vegetables, as well as the detection of *Cryptosporidium* spp. in strawberries and raspberries [40, 41]. Food as a vehicle of infection and a source of infection warrants further investigation.

## Conclusions

Cryptosporidiosis is the most common notifiable domestic parasitic disease in Sweden. The national microbiological surveillance programme is important and has led to increased knowledge and awareness of human cryptosporidiosis in Sweden. *Cryptosporidium parvum* was shown to be the dominant species causing disease, as well as outbreaks, of which only foodborne outbreaks and outbreaks associated with direct animal contact were detected. A high diversity of species and subtypes, as well as diversity within the same subtype was detected. Importantly, cryptosporidiosis is not primarily a paediatric disease in Sweden, but appears to affect adults to a great extent.

### Electronic supplementary material

Below is the link to the electronic supplementary material.

Supplementary Material 1

## Data Availability

No datasets were generated or analysed during the current study.
