# Supplementary material for: Cryptosporidium species and subtypes identified in human domestic cases through the national microbiological surveillance programme in Sweden from 2018 to 2022
Source: BMC Infect Dis. 2024 Jan 30;24:146. doi: 10.1186/s12879-024-09049-x (PMC10826111; doi:10.1186/s12879-024-09049-x)
Supplement: Supplementary file 1 — Supplementary Material 1 [file 12879_2024_9049_MOESM1_ESM.docx]

**Supplementary Fig. 1.** Notification rate of domestic cryptosporidiosis cases from 2018 to 2021 based on age groups in Sweden compared to the average notification rate in EU/EEA (Data from Surveillance Atlas of Infectious Diseases).
